# Improving the representativeness of influenza viruses shared within the WHO Global Influenza Surveillance and Response System

**DOI:** 10.1111/irv.12362

**Published:** 2016-02-09

**Authors:** Dmitriy Pereyaslov, Galina Zemtsova, Christine Gruessner, Rodney S. Daniels, John W. McCauley, Caroline S. Brown

**Affiliations:** ^1^ Division of Communicable Diseases, Health Security & EnvironmentWHO Regional Office for EuropeCopenhagen ØDenmark; ^2^ WHO Collaborating Centre for Reference and Research on InfluenzaThe Francis Crick InstituteMill Hill LaboratoryLondonUK

**Keywords:** Influenza vaccine virus selection, influenza vaccine viruses, WHO recommendations

## Abstract

**Background:**

Sharing influenza viruses within the WHO Global Influenza Surveillance and Response System is crucial for monitoring evolution of influenza viruses.

**Objectives:**

Analysis of timeliness and geographic representativeness of viruses shared by National Influenza Centres (NICs) in the WHO European Region with the London WHO Collaborating Centre for Reference and Research on Influenza for the Northern Hemisphere's 2010–2011 and 2011–2012 influenza seasons.

**Materials and methods:**

Data from NICs on influenza‐positive specimens shared with WHO CC London for the above‐mentioned influenza seasons were analyzed for timeliness of sharing with respect to the February deadline (31 January) for inclusion in the WHO consultations on the composition of influenza virus vaccines for the Northern Hemisphere and geographic representativeness.

**Results:**

The 2010–2011 and 2011–2012 seasons were different in terms of the seasonal pattern, the timing of the epidemic, and the dominant virus. Consistent patterns of virus sharing across the seasons were observed. Approximately half the viruses collected before the deadline were not shared within the deadline; the average delay between date of specimen collection and shipment receipt was 3 and 1·5 months for the first and second season, respectively.

**Conclusion:**

A baseline was provided for future work on enhancement of specimen sharing in the WHO European Region and improving the vaccine virus selection process. Greater insight into virus selection criteria applied by countries and the causes of delays in shipment are needed to understand the representativeness of viruses shared and to assess the importance of this for vaccine strain selection.

## Introduction

Influenza A and B viruses evolve at high rates with mutations being prevalent in their hemagglutinin genes encoding glycoprotein (HA), the major target for neutralizing antibodies.^1^ Amino acid substitutions in antibody‐binding sites cause antigenic drift, which, in its turn, results in the occurrence of seasonal influenza epidemics of different severity.^2^, ^3^ To overcome antigenic drift, continuous virologic and disease surveillance is undertaken to ensure that viruses included in seasonal influenza vaccines and circulating viruses are antigenically similar. The World Health Organization (WHO) monitors evolution of influenza viruses through the activities of the Global Influenza Surveillance and Response System (GISRS).^4^ A core function of the GISRS is the sharing of seasonal influenza viruses by WHO‐recognized National Influenza Centres (NICs) with WHO Collaborating Centres for Reference and Research on Influenza (WHO CCs). The WHO CCs conduct detailed antigenic and genetic characterization of the viruses to inform recommendations on the composition of influenza vaccines for use in the relevant subsequent season. Recommendations are made twice yearly at WHO consultations on the composition of influenza virus vaccines (VCM) and at least 6 months before the influenza season to meet strict timetables relating to the provision of candidate vaccine viruses, national regulatory authorities decision‐making, vaccine production, validation, licensing, and distribution.^5^ On average, at least one vaccine virus is substituted every one or two years.^6^ Additionally, data generated by the GISRS contribute to antiviral susceptibility monitoring, assessing the risk posed by novel influenza viruses with pandemic potential, and diagnostic development. In the WHO European Region, 64 national influenza laboratories are active in 50 countries, of which 52 laboratories in 41 countries are WHO‐recognized NICs. All participate in EuroFlu, the WHO Regional Office for Europe platform for influenza surveillance.^7^

National Influenza Centres receive clinical specimens collected from patients during the influenza season and perform initial identification and virus isolation. To contribute effectively to the VCM, WHO provides guidance on the selection and number of viruses which should be shared with the WHO CCs.^8^

The majority of NICs in the WHO European Region share viruses with the WHO Collaborating Centre for Reference and Research on Influenza at the National Institute for Medical Research (currently the Francis Crick Institute), London, United Kingdom (WHO CC London). Every shipment of viruses to WHO CC London includes a standard form requesting virologic, epidemiologic, and patient‐related information for specimens shipped. These forms constitute a standardized dataset allowing the first systematic review of the representativeness of seasonal influenza‐positive specimens shared within GISRS to be conducted, together with an assessment of the compliance with/utility of the WHO guidance. Given the limitation of resources available for public health, and the need for effective implementation of the pandemic influenza preparedness (PIP) framework for the sharing of influenza viruses and access to vaccines and other benefits, evaluating this crucial GISRS function is essential.^9^

## Materials and methods

Data provided by NICs with influenza‐positive specimens shared with WHO CC London for two Northern Hemisphere influenza seasons, 2010–2011 (week 40/2010 to week 39/2011) and 2011–2012 (week 40/2011 up to week 28/2012), were analyzed with regard to the number of viruses shared, geographic origin, virus identity (influenza type, A subtype, B lineage and strain name), and the timeliness of sharing with respect to the February VCM deadline (comparing specimen collection dates and shipment receipt dates at WHO CC London to assess delays). Sample selection criteria in the WHO guidance require that shared viruses should be representative of influenza activity in each country for different age groups, clinical settings, geographic locations, different phases of progression of the epidemic, and epidemiologic indicators (e.g., severity of illness). This guidance additionally stipulates the timeliness in which shipments should be made to a WHO CC; viruses received before the third week of January and before the third week of August can be characterized in time for February and September VCMs, respectively. Timeliness was evaluated based on the date of shipment receipt by WHO CC London. The deadline for receipt of specimens in order for them to be characterized in time for the February VCM was set at 31 January (week 5) for both seasons. This deadline is two weeks beyond the WHO guidance, being extended in order to accommodate more shipments from NICs in the WHO European Region. Shipment delays for both seasons were calculated as the mean time in days between specimen collection date and shipment receipt by WHO CC London.

In addition, the distribution of influenza type and subtype among viruses shared with WHO CC London was compared with the virus detection totals reported to EuroFlu during the two seasons.

To analyze geographic representativeness, WHO European Region Member States were grouped into subregions according to United Nations geographic definitions which groups countries into southern, northern, eastern, and western Europe, as well as central and western Asia.^10^ To determine the representativeness of shared viruses in relation to timing of an influenza season, peak seasonal influenza activity for the whole region was determined by summing of combined sentinel and non‐sentinel specimens positive for influenza viruses A and B and graphical analysis of outpatient surveillance for influenza‐like illness (ILI) and/or acute respiratory infection (ARI), according to data reported to EuroFlu. Peak weeks were defined as those with the highest laboratory‐confirmed influenza positivity and ILI/ARI rates. Median influenza peak weeks were calculated for each country, subregion and the region.

## Results

### Timing of the 2010–2011 and 2011–2012 influenza seasons and numbers of viruses shared

**Figure 1:**
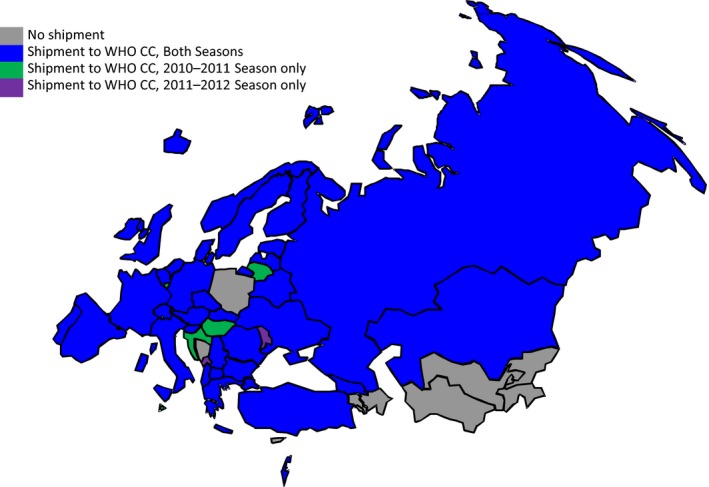
Countries sharing viruses with WHO CC London in 2010–2011 (38 countries, *n* = 1837) and 2011–2012 influenza seasons (35 countries, *n* = 1117).

The 2010–2011 season started slightly earlier than the 2011–2012 season, and previous seasons, and it progressed from west to east across much of the WHO European Region, while there was no distinct pattern in spread observed in 2011–2012. Influenza activity peaked in weeks 4/2011 and 8/2012 for the two seasons studied. During the 2010–2011 influenza season, 1837 specimens were shared by NICs in 39 of 53 Member States with WHO CC London, and for 2011‐2012, 1117 viruses were shared by NICs in 35 Member States. For the two seasons combined, this represents 2954 specimens shared by 41 Member States (Figure 1). The number of viruses shared by any one Member States ranged from 5 to 207 (median 31·5) in the 2010–2011 season and from 1 to 128 (median 28) in the 2011–2012 season. In terms of data completeness, there was complete reporting for laboratory ID, specimen type, and virus identity variables. During the 2010–2011 season, data on age were available for 1620 (88%) submitted specimens and gender was specified for 1247 (68%). During the 2011–2012 season, the specimen collection date was reported for 1074 (96%) submitted specimens, with age and gender data available for 864 (77%) and 887 (79%), respectively. Clinical setting (severity) was the most underreported category for both seasons, with hospital or outpatient setting indicated for 659 (36%) and 656 (59%) specimens in consecutive seasons.

### Timeliness of influenza viruses shared with respect to the February VCM deadline

**Figure 2:**
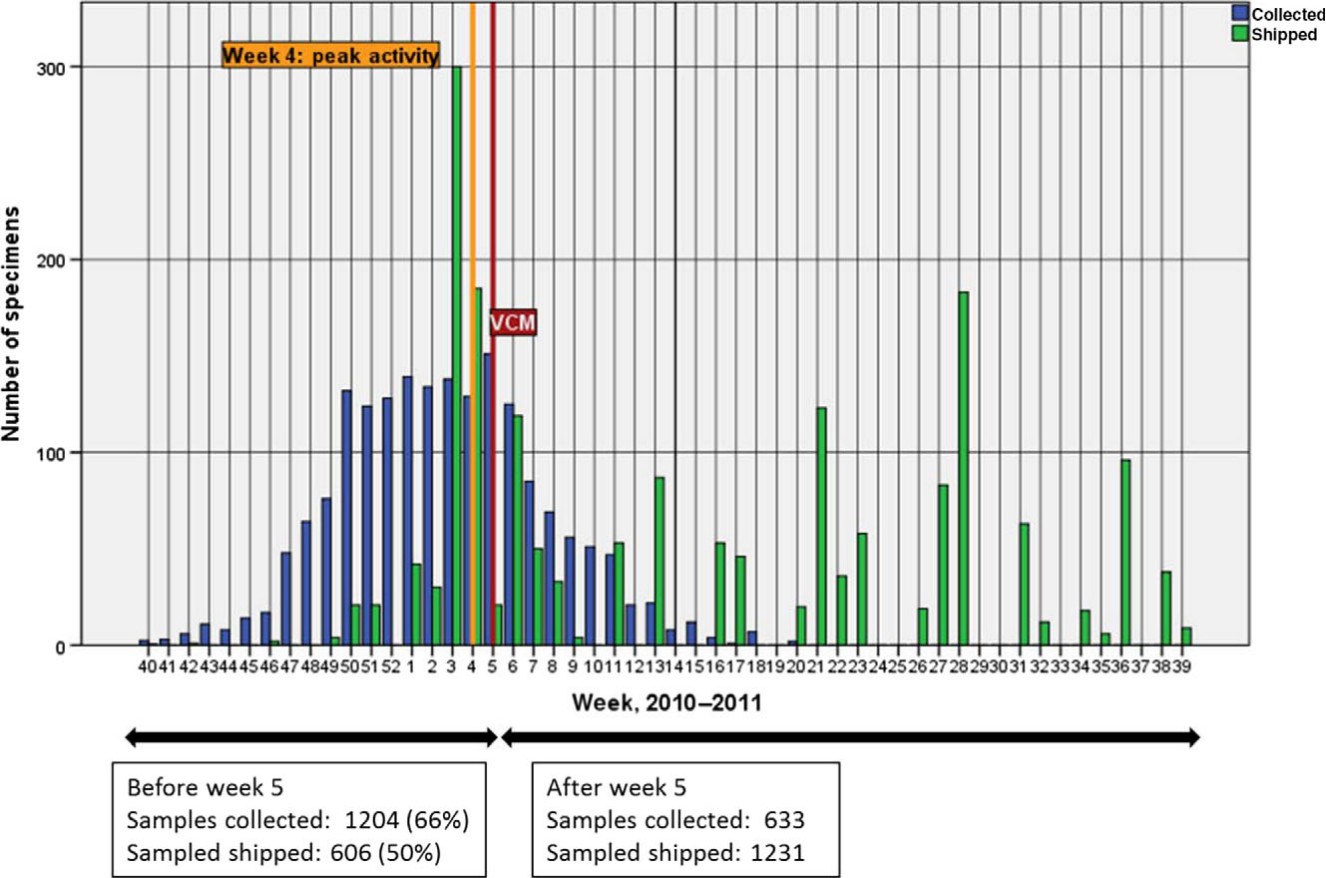
Specimens shared with WHO CC London, 2010‐2011 (38 countries, n=1837).

Of 1837 specimens received by WHO CC London during the 2010–2011 season, 1204 (66%) had collection dates before 31 January 2011 and 606 (50%) of these (from 24 Member States) were received before the February VCM deadline (Figure 2). The mean time between specimen collection date and shipment receipt was 90·3 days and varied considerably by subregion from 59 days for western Europe to 181 days for central Asia (Table 1).

**Table 1:**
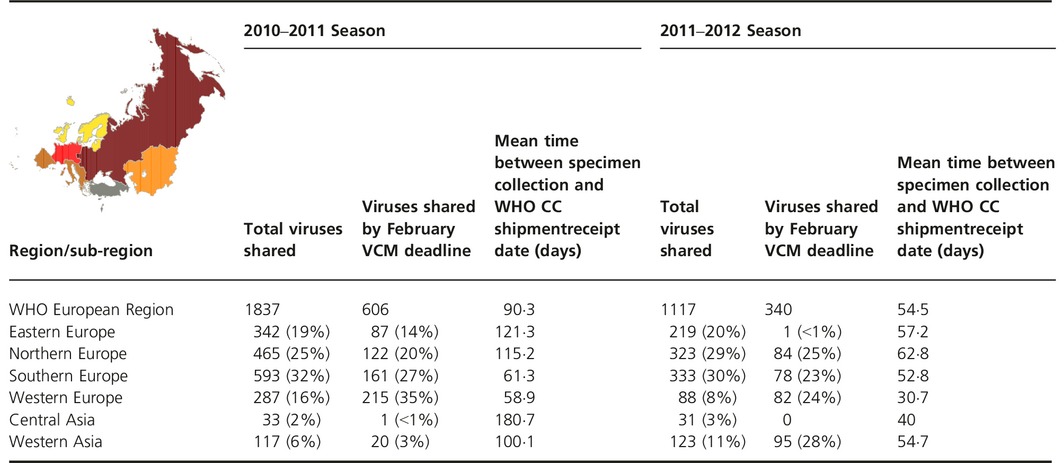
Number of specimens received by WHO CC London, 2010–2011 and 2011–2012 seasons for the WHO European Region and subregions

**Figure 3:**
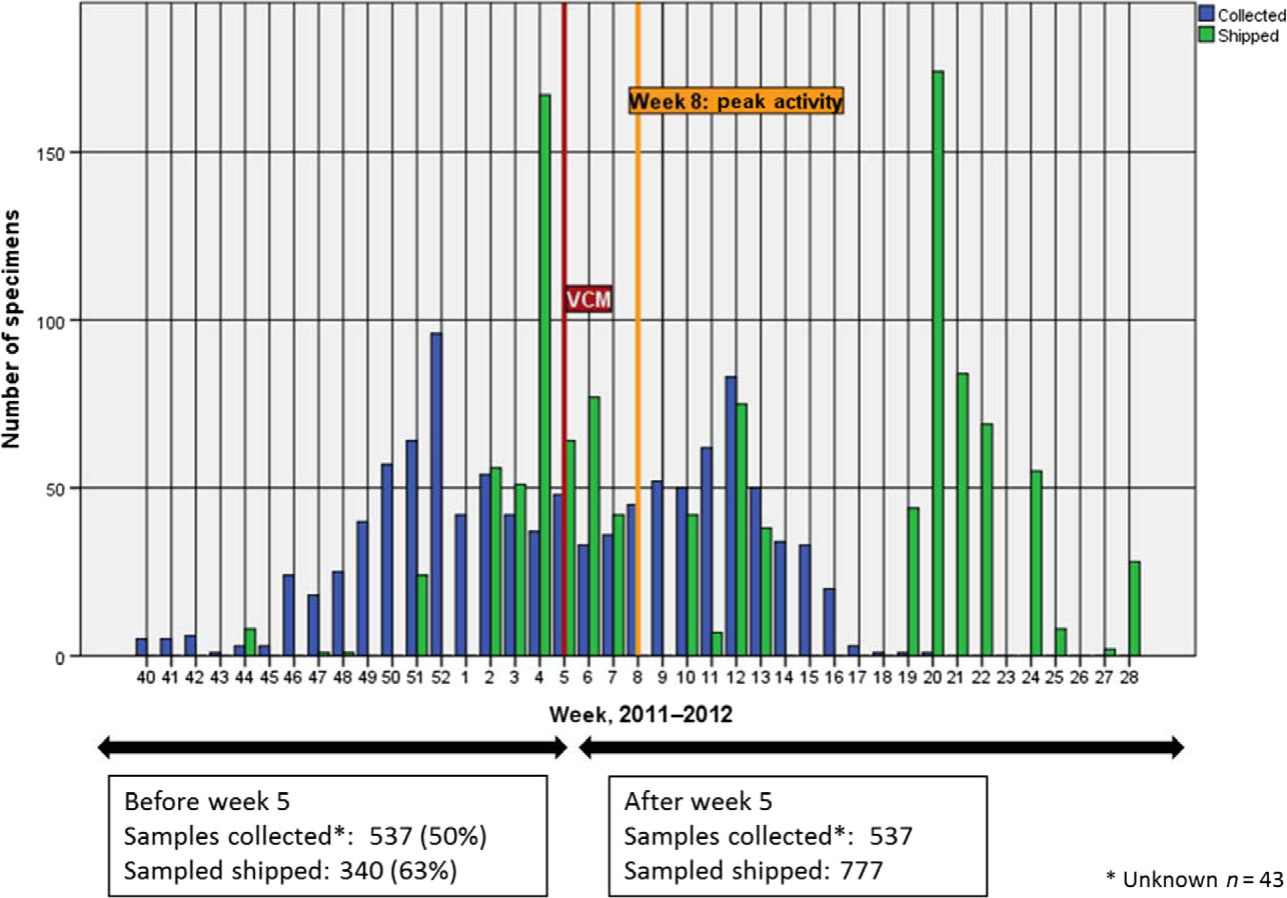
Specimens shared with WHO CC London, 2011–2012 (35 countries, *n *= 1117).

A similar pattern was observed in the 2011–2012 season: of 1117 specimens received by WHO CC London, 537 (48%) had collection dates before 31 January 2012 with 340 (63%) of these, from 18 Member States, being received before the February VCM deadline (Figure 3). The mean time between specimen collection date and shipment receipt was 54·5 days and varied considerably by subregion from 31 days for western Europe to 63 days for northern Europe (Table 1). Specimen collection date was missing for 43 specimens and these were excluded from the analysis.

### Geographic representativeness by subregion of influenza viruses shared over the whole season and with respect to the February VCM deadline

In the 2010–2011 season, four subregions (southern, northern, eastern, and western Europe) contributed 92% of specimens shared. By the February VCM deadline, these same four subregions contributed 96% of specimens shared. Western and central Asia subregions accounted for 21/606 (3·5%) specimens shared by the deadline (Table 1).

During the 2011–2012 season, three subregions (southern, northern, and eastern Europe) shared the majority (78%) of specimens. However, western Asia contributed 28% of the 340 specimens shared before the February VCM deadline with northern, western, and southern Europe contributing the remainder but for one specimen received from eastern Europe. No specimens were received from central Asia (Table 1).

Across both seasons, southern, northern, and eastern Europe shared the greatest numbers of specimens with WHO CC London. However, while western Europe contributed 16% and 8% of shared specimens in the consecutive seasons, their specimens accounted for 35% and 24% of specimens submitted in time for the respective February VCMs. While eastern Europe shared specimens consistently, 19% and 20% for the two seasons, only 87/342 (25%) and 1/219 (0·5%) were received in time for the respective February VCMs. Central Asia was most underrepresented for both seasons, accounting for 2% and 3% of shared specimens in the consecutive seasons, but only one specimen was received before the February VCM deadline in 2011, and none before the deadline in 2012. Western Asia contributed 6% and 11% of shared specimens during the 2010–2011 and 2011–2012 seasons and the number of viruses meeting the February VCM deadlines was 20/117 (17%) and 95/123 (77%), respectively, demonstrating the greatest increase in timely sharing among all subregions.

### Representativeness of specimens shared compared to circulating virus type/subtype in the WHO European Region as reported to EuroFlu

**Figure 4:**
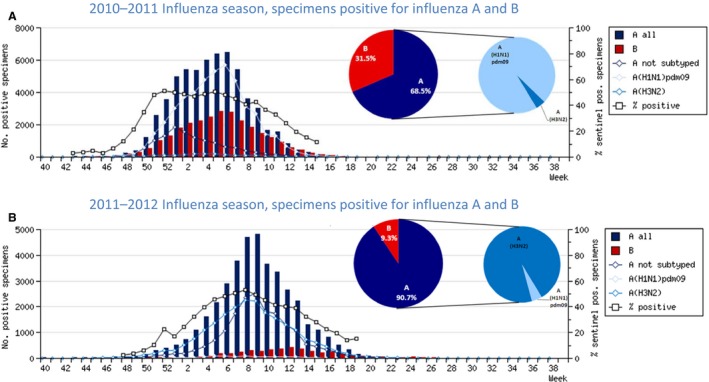
2010–2011 and 2011–2012 Influenza seasons in the WHO European Region as reported to EuroFlu.

A(H1N1)pdm09 viruses predominated in 2010–2011, co‐circulating with influenza B, with low numbers of A(H3N2) viruses detected. In contrast, A(H3N2) viruses predominated in 2011–2012 with low numbers of influenza B viruses and negligible A(H1N1)pdm09 virus detections (Figure 4).

Specimens shared with WHO CC London in 2010–2011 were 62·8% influenza type A and 37·2% influenza type B positive, corresponding to influenza activity in the WHO European Region being associated with influenza types A (68·5%) and B (31·3%) viruses, which co‐circulated in most Member States. Influenza A predominated in most Member States in the region in the initial phase of the epidemic, with influenza B becoming dominant in the region by end of season (Figure 4). Influenza A(H1N1)pdm09 was identified as the dominant subtype among specimens shared with WHO CC London (49·1%) and circulating in the region (52·2%). Of the influenza type B viruses, a higher proportion of B/Victoria lineage viruses were shared with WHO CC London compared to B/Yamagata lineage viruses, reflecting the pattern observed in circulating viruses that season.

During the 2011–2012 season, the specimens shared with WHO CC London were 86·5% and 13·5% positive for influenza types A and B, respectively, again reflecting the influenza activity in the WHO European Region: 90·7% type A and 9·3% type B influenza viruses, which co‐circulated in most Member States (Figure 4). Influenza A(H3N2) was identified as the dominant subtype among specimens shared with WHO CC London (77·2%) and circulating in the region (48·0%). Of the influenza type B viruses, equal proportions of influenza B/Victoria (4·4%) and B/Yamagata (3·5%) lineage viruses were shared with WHO CC London, reflecting the pattern observed among circulating viruses.

For both seasons, the proportions of influenza type A subtype and B lineage specimens shared with WHO CC London by the VCM deadline corresponded with the relative proportions detected among viruses circulating in the region.

## Discussion and conclusions

National Influenza Centres in the WHO European Region made significant contributions to WHO recommendations on seasonal influenza vaccine virus selection during the two influenza seasons analyzed by sharing 2954 influenza‐positive specimens with WHO CC London. Of these, 946 were shared in time for the February VCMs. The viruses shared were representative of circulating influenza viruses in different geographic subregions of the WHO European Region. However, about half the specimens with collection dates before the February VCM deadline (31 January) were not shared in time for the February VCM. Shipments often occurred weeks or months after sample collection dates and large subregions of the WHO European Region (eastern Europe, central Asia, and western Asia), accounting for more than 50% of the region's population, were underrepresented.

The WHO European Region is geographically extensive and seasonal influenza epidemics peak at different times in different geographic subregions.^11^, ^12^, ^13^ Lack of timely sharing can partly be explained by the fact that influenza seasons peak either concomitantly with or after the February VCM shipment deadline; this would prevent countries where the influenza season starts later from shipping specimens in time. This is a probable factor for eastern Europe and western Asia subregions where the timings of the 2010–2011 and 2011–2012 seasons were later than in the rest of the region. If NICs are unable to ship specimens collected before the February VCM deadline, these viruses are very valuable and may still be shipped to allow characterization in time for the September VCM. However, all NICs should collect and share late season influenza‐positive specimens, that is, more recent viruses with respect to the September VCM. Had the February VCMs taken place two weeks later during the two seasons studied, the number of viruses shared in a timely manner would have increased by 10% and 20%, respectively.

Variation in timing of the season can only partially explain the lack of timely sharing. Another issue was that the average delay between date of specimen collection and shipment receipt, 3 and 1·5 months for the first and second season, respectively, although this varied significantly by subregion with northern Europe and central Asia having the longest delays. As VCM recommendations rely on recently circulating viruses, factors influencing this delay, which could be internal or external to the NIC, should be investigated. One of the internal factors could be difficulties in virus isolation reported recently especially for A(H3N2) viruses in Madin‐Darby canine kidney (MDCK) cells, attributable to a loss of receptor binding capability of the H3‐HA over recent years^14^. The timeliness of sharing could be improved if NICs were encouraged to ship influenza‐positive clinical specimens, if there was insufficient time for or difficulties with virus isolation within the NIC. It was not possible to adjust the timeliness analysis for delays between clinical specimen collection, receipt by NIC, and further processing for identification of virus type/subtype/lineage and virus isolation as this information was not collected for specimens shipped.

Of the external factors, it is important to note that in 2011–2012, NICs could send only one shipment covered by the WHO Shipment Fund (except for NICs in low and lower‐middle income Member States), which could have led to a shift toward shipping at the end of the influenza season. While the effects of the WHO Shipment Fund restrictions on shipping patterns were not investigated by this study, it is notable that there was only a slight decrease, 606/1837 (33%) and 340/1117 (30%), in the proportion of specimens shared in a timely manner for the two seasons (Table 1).

Another factor is that twelve Member States in the WHO European Region do not have a WHO‐recognized NIC and are therefore not bound by the Terms of Reference for National Influenza Centres^15^ to forward viruses to a WHO CC. Some of these laboratories share viruses with WHO CC London as part of their involvement in the WHO Regional Office for Europe influenza surveillance activities. In addition, WHO European Region NICs in three Member States shared specimens with WHO CC London and another WHO CC during the two seasons and one NIC shared viruses with another WHO CC only.

Although reports from WHO consultations for Improving Influenza Vaccine Virus Selection highlighted the increasing coverage of influenza surveillance in the world^16^, ^17^, ^18^ with more NICs in all WHO regions sharing viruses with WHO CCs, this review is the first in‐depth analysis of virus sharing within GISRS with a view to determining the extent of the representativeness of viruses shared by the WHO European Region, broken down by geographic subregions, and assessing the extent to which virus sharing practices within GISRS adhere to WHO guidance. The two Northern Hemisphere influenza seasons analyzed were different in terms of season pattern, timing of the epidemic, and dominant virus. Consistent patterns of sharing across the two diverse influenza seasons studied were observed. The analysis provides a baseline for the future work of WHO consultation to improve the WHO vaccine virus selection process and guide enhancement of specimen sharing in the WHO European Region and globally.

This review focused on geographic and timeliness aspects of representativeness. The true extent of demographic and epidemiologic representativeness of specimens shared within GISRS could not be determined due to incomplete/missing data, particularly for patient age and clinical settings (severity of illness) variables. Also, WHO guidance may need to be reviewed to emphasize the need for NICs to share the most recently collected specimens/viruses. Further, efforts to encourage NICs to collect and provide complete virologic and epidemiologic surveillance data should be made. More complete data as well as greater insight into virus selection criteria by NICs and factors influencing timely shipments are needed. These should be further explored to fully understand the representativeness of viruses being shared within GISRS and the reasons for viruses not being shipped in time for the February VCM, as well as implications for vaccine virus selection.

## Disclaimer

The authors alone are responsible for the views expressed in this article and they do not necessarily represent the views, decisions, or policies of the institutions with which they are affiliated.

The boundaries and names shown and the designations used in this publication do not imply the expression of any opinion whatsoever on the part of the World Health Organization concerning the legal status of any country, territory, city, or area or of its authorities, or concerning the delimitation of its frontiers or boundaries.
